# The Effects of Medicinal Plants and Bioactive Natural Compounds on Homocysteine

**DOI:** 10.3390/molecules26113081

**Published:** 2021-05-21

**Authors:** Mohammad Amin Atazadegan, Mohammad Bagherniya, Gholamreza Askari, Aida Tasbandi, Amirhossein Sahebkar

**Affiliations:** 1 Department of Clinical Nutrition, School of Nutrition and Food Science, Isfahan University of Medical Sciences, Isfahan 8174673461, Iran; atazadegan95@gmail.com; 2 Food Security Research Center, Isfahan University of Medical Sciences, Isfahan 8174673461, Iran; Askari@mui.ac.ir; 3 Anesthesia and Critical Care Research Center, Isfahan University of Medical Sciences, Isfahan 8174673461, Iran; 4 Department of Community Nutrition, School of Nutrition and Food Science, Isfahan University of Medical Sciences, Isfahan 8174673461, Iran; 5 Applied Biomedical Research Center, Mashhad University of Medical Sciences, Mashhad 9177948564, Iran; aidatasbandi@yahoo.com; 6 Biotechnology Research Center, Pharmaceutical Technology Institute, Mashhad University of Medical Sciences, Mashhad 9177948564, Iran; 7 School of Pharmacy, Mashhad University of Medical Sciences, Mashhad 9177948954, Iran

**Keywords:** non-communicable diseases, cardiovascular disease, homocysteine, herbal medicine, medicinal plants

## Abstract

Background: Among non-communicable diseases, cardiovascular diseases (CVDs) are the leading cause of mortality and morbidity in global communities. By 2030, CVD-related deaths are projected to reach a global rise of 25 million. Obesity, smoking, alcohol, hyperlipidemia, hypertension, and hyperhomocysteinemia are several known risk factors for CVDs. Elevated homocysteine is tightly related to CVDs through multiple mechanisms, including inflammation of the vascular endothelium. The strategies for appropriate management of CVDs are constantly evolving; medicinal plants have received remarkable attention in recent researches, since these natural products have promising effects on the prevention and treatment of various chronic diseases. The effects of nutraceuticals and herbal products on CVD/dyslipidemia have been previously studied. However, to our knowledge, the association between herbal bioactive compounds and homocysteine has not been reviewed in details. Thus, the main objective of this study is to review the efficacy of bioactive natural compounds on homocysteine levels according to clinical trials and animal studies. Results: Based on animal studies, black and green tea, cinnamon, resveratrol, curcumin, garlic extract, ginger, and soy significantly reduced the homocysteine levels. According to the clinical trials, curcumin and resveratrol showed favorable effects on serum homocysteine. In conclusion, this review highlighted the beneficial effects of medicinal plants as natural, inexpensive, and accessible agents on homocysteine levels based on animal studies. Nevertheless, the results of the clinical trials were not uniform, suggesting that more well-designed trials are warranted.

## 1. Introduction

**Figure 1:**
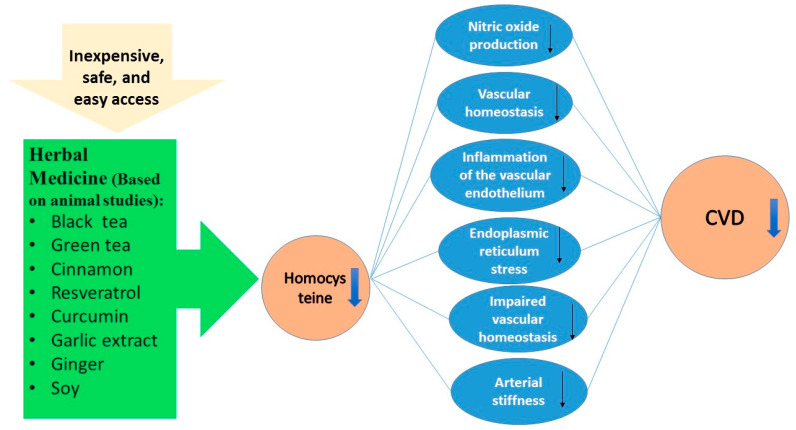
A schematic summary of pathways depicting the beneficial effects of herbal medicine on serum homocysteine (based on animal studies) and the possible mechanisms occurred after reduction in homocysteine level resulted in favorable outcomes; reduction in cardiovascular diseases (CVD).

In recent decades, we have experienced a shift in the disease burden towards non-communicable diseases, which is likely due to the improved efficacy of treatments and lifestyle changes [1,2]. According to the World Health Organization (WHO), non-communicable diseases account for 75% of global deaths in 2020; of these, cardiovascular diseases (CVDs) are the leading cause of mortality and morbidity in communities [3,4,5]. By 2030, CVD-related deaths are projected to reach a global increase of 25 million [6,7]. The documented studies show that non-communicable diseases are responsible for $7.28 trillion loss from 2011 to 2025, half of which is related to CVDs. It is estimated that a 10% reduction in CVDs will reduce the economic loss by $378 billion over the years [8,9]. The known risk factors of CVDs include obesity, smoking, alcohol consumption, hyperlipidemia, hypertension, hyperhomocysteinemia, among others [10,11]. Homocysteine is a mediator in methylation cycle that acts as a cysteine and methionine precursor, a substrate for tissue folate recycling, and methyl receptor in choline catabolism [12,13,14]. Methionine is first converted to S-adenosylmethionine (SAM), which is, in turn, converted to S-adenosyl homocysteine (SAH) in a reversible reaction. Subsequently, S-adenosine homocysteine breaks down into adenosine and homocysteine. In all tissues, 5-Methyltetrahydrofolate (as methyl group donor; in the liver, betaine acts as a methyl donor) and methylcobalamin (as coenzyme) are required to convert homocysteine to methionine with the help of vitamin B12. Homocysteine itself is first converted to cystathionine and then to cysteine (with the help of vitamin B6), which is finally excreted in the urine as sulfur-containing compounds or converted to taurine [15,16,17]. Homocysteine and its derivatives are bound to intracellular proteins and their presence in the blood may be due to their removal from the cells to be used by other tissues [12,18,19]. In 1962, the association between homocysteine and CVDs was established [20]. The normal homocysteine levels are considered to be less than 10 micrograms per liter. Hyperhomocysteinemia is defined as mild (12–30 micrograms per liter), intermediate (31–100 micrograms per liter), and severe (more than 100 micrograms per liter) [21,22]. In the general population, the prevalence of hypercysteinemia is approximately five to 10 percent and 30 to 40 percent in the elderly [23]. In population studies, an inverse relationship was found between homocysteine concentrations and vitamin B12 or abdominal aortic diameter in the elderly [24,25]. Several meta-analyzes have shown that hyperhomocysteinemia is a strong predictor of CVDs [26,27,28,29,30]. A five micromole per liter increase in blood homocysteine levels is associated with a 32% increase in myocardial ischemia and a 59% increase in myocardial infarction [31]. Additionally, wild-type and mutant methyltetrahydrofolate increase the risk of cardiovascular disease by 25% and the homocysteine level by 16% [31]. Although the mechanism of action of homocysteine on blood vessels is not fully understood, it is thought to damage different layers of arteries [32]. Hyperhomocysteinemia has negative impacts on endothelial cells by affecting the production of nitric oxide, which regulates vascular tone [33,34,35,36,37,38]. It may also impair the maintenance of vascular homeostasis by interfering with the hydrogen sulfide signaling pathway that is closely related to nitric oxide [39]. Evidence suggests that hyperhomocysteinemia promotes inflammation of vascular endothelium by inducing inflammatory factors, such as interleukin-6, interleukin-8, and tumor necrosis factor alpha [40,41,42]. It is shown that high levels of homocysteine are associated with increased endoplasmic reticulum stress, which, in turn, leads to vascular inflammation [43] and oxidative stress in mice [44]. Additionally, homocysteine causes endothelial cell apoptosis, which is a primary sign of atherosclerosis [44,45]. In a study conducted in 2015, it was indicted that homocysteine metabolites (homocysteine thiolactone and N-homocysteine) could disrupt vascular homeostasis [46]. Another effect of homocysteine on the dysregulation of vascular homeostasis is through impairing the function of smooth muscle cells and inducing their proliferation [47,48,49,50]. Homocysteine causes arterial stiffness by the deposition of collagen in the vascular wall through the induction of connective tissue growth factor (CTGF). It also triggers the formation of aneurysm in coronary arteries by inducing the production of elastin degrading enzymes [51,52,53,54]. The preventive efforts are the best way of managing CVDs, and they are achieved by a proper lifestyle, including a balanced diet, and adequate physical activity [55], which may be not be favorable to some individuals. Although the strategies for the appropriate treatment of CVDs are constantly evolving; they are often ineffective and could have serious side effects [55,56]. Hence, the recent focus is on natural or low-risk supplements, such as herbal medicines or functional foods [57]. The protective effects of nutraceuticals on CVDs have been previously identified [58,59]. The term nutraceutical was first coined in 1989 by Stephen Deflis. Nutraceuticals are defined as “foods or parts of a food that provide medical or health benefits including prevention or treatment of a disease” [60]. Nutraceuticals are substances that can be used instead of a medication as dietary supplements to control, prevent, or even treat diseases, such as CVDs [61]. These are known as food drugs and they are particularly helpful in patients who do not have access to a medication or for treatment of chronic diseases, such as osteoporosis, heart diseases, among others [62]. Recently, medicinal plants are becoming the focus of several researches, since these natural products have shown promising effects on the prevention and treatment of various diseases, particularly CVDs [63,64,65,66], diabetes mellitus [67,68,69], hypertension [70,71,72], and non-alcoholic fatty liver disease (NAFLD) [56]. The beneficial effects of herbal bioactive compounds on 3-hydroxy-3-methyl-glutaryl-coenzyme A reductase (HMG-CoA reductase) [73], Apolipoprotein B (Apo B) [74], and small dense LDL [74] have recently been reviewed. These are three important factors with unfavorable effects on CVDs. The effects of nutraceuticals and herbal products on CVDs/dyslipidemia have been previously studied. However, to our knowledge, the association between herbal bioactive compounds and homocysteine has not been reviewed in detail [63,64,65,66,75,76,77,78,79]. Thus, the main objective of this study is to address the efficacy of bioactive natural compounds on homocysteine levels, which was assessed through clinical trials and animal studies (Figure 1).

## 2. Results

### 2.1. Plants

#### 2.1.1. Black Tea

Tea is widely consumed around the world, and all the popular types of tea, like black tea, are manufactured from the leaves of *Camellia sinensis* (L.) Kuntze from Theaceae family. Several studies have shown that tea and its bioactive polyphenolic constituents have numerous beneficial effects on the prevention of diseases, like cancer, diabetes, arthritis, CVDs, stroke, and obesity [80,81,82,83]. These effects are due to antioxidative, anti-inflammatory, antihypertensive, cholesterol-lowering, antimicrobial, anticarcinogenic, neuroprotective, and thermogenic properties of the tea [83]. The favorable effects of tea on CVDs have been demonstrated in epidemiological studies and clinical trials [83]. Its effect on homocysteine level is one proposed mechanism. In one experiment, the rats were assigned into three groups: (1) Vehicle (saline), (2) angiotensin (Ang) II (50 ng/kg/min.) to induce hypertension, and (3) Ang II + Black tea extract (BT) in which animals were given a 15 mg/kg/day of black tea extract (starting from Day 1 after Ang II pump insertion) for two weeks. Initially, angiotensin II infusion enhanced the plasma homocysteine level and it resulted in endoplasmic reticulum (ER) and oxidative stress, which, in turn, triggered endothelial dysfunction. However, black tea extract dramatically decreased the blood pressure and plasma homocysteine and, consequently, protected arteries of hypertensive rats from ER stress and endothelial dysfunction [84]. According to a clinical trial conducted by Hodgson et al., 20 adults with CAD were randomly assigned to four groups: (I) water and no meal, (II) black tea and no meal, (III) meal with water, or (IV) meal with black tea. The participants were asked to drink a cup of black tea (contained 2.2 g of tea leaves at times 0, 1.5 h, and 3 h) or three cups of hot water with and without a meal (comprised a sausage, egg, bacon, McMuffins, and two hash browns). The total homocysteine was measured at baseline and 3.5 h after drinking black tea or hot water with and without a meal. In the end, an acute increase in homocysteine was observed after drinking black tea. Although the meal caused an acute decline in homocysteine level, it did not alter the homocysteine-raising effect of tea [85]. Similarly, in a randomized crossover study, 22 subjects were divided into two groups to drink 1250 mL black tea/d (five cups each containing 2 g tea leaves in 250 mL boiled water) or 1250 mL hot water/d for four weeks. For the next four weeks, the participants consumed the alternate drink. The findings demonstrated that black tea did not significantly alter the mean homocysteine concentrations [86]. Twenty healthy subjects in another crossover study received a diet low in polyphenols and they were randomly assigned into four groups of supplemented regimens: (I) 2 g chlorogenic acid (a compound found in coffee and black tea), (II) 4 g black tea solids (III) 440 mg quercetin-3-rutinoside, or (IV) 0.5 g citric acid as a placebo. The duration of each trial was seven days (a four-week trial). The results showed that, after 4–5 h of supplementation, chlorogenic acid and black tea both raised total homocysteine concentrations in plasma when compared to the placebo. Quercetin-3-rutinoside exerted no effect on plasma homocysteine [87]. The different results between the animal and clinical studies might be due to the fact that, in a previous animal study, black tea was administered in experimentally hypertensive rats. It was suggested that homocysteine levels are reduced after the intake of black tea polyphenols in hypertension, which might be mediated and attributed to the promotion of homocysteine metabolism [84]. On the other hand, in the clinical trials, a small sample size of healthy subjects without hypertension were recruited, which might be a potential explanation for reporting the different results between animal and human studies [86,87]. In addition, it is suggested that tea and coffee, and their major constituents, such as polyphenols and caffeine, might increase homocysteine by acting as acceptors of methyl groups during the metabolism of methionine to homocysteine [86,87,88,89]. When considering these controversial findings and the fact that several studies suggest that tea consumption has a protective role against CVDs [90,91,92], there have been calls to conduct more studies in the future related to both the mechanistical and clinical aspects to explore the role of black tea on homocysteine.

#### 2.1.2. Green Tea

**Figure 2:**
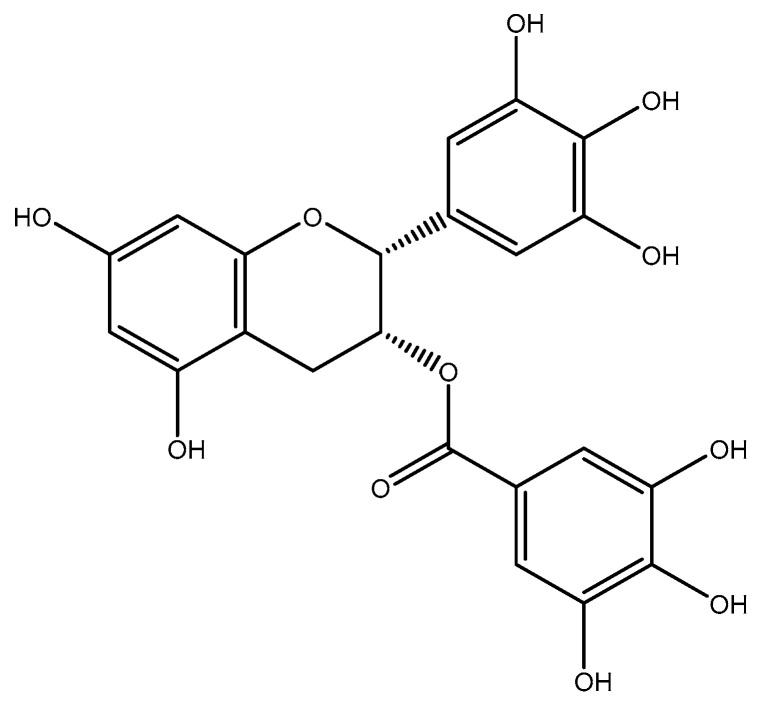
Chemical structure of (-)-epigallocatechin gallate.

Green tea is a non-fermented tea [93] that is traditionally used as a natural medicine. It is a rich source of polyphenols, mainly epigallocatechin-3-gallate (EGCG) (Figure 2) [94,95], which is proved to have favorable effects on neurological diseases, cancer, inflammation [94,96,97,98,99], and homocysteine-induced cerebrovascular injury [100]. In a study on adult male Wister rats, six groups were randomly assigned: (1st) normal laboratory diet, (2nd) 2.5 mg/kg body weight EGCG, (3rd) 5 mg/kg body weight EGCG, (4th) exposure to 4 Gy of γ radiation, (5th) 2.5 mg/kg body weight EGCG + exposure to 4 Gy of γ radiation, and (6th) 5 mg/kg body weight EGCG + exposure to 4 Gy of γ radiation. The intervention was performed for three consecutive days in the 2nd and 3rd groups, and for two days in 5th and 6th groups. The third dose was administered for 30 min. before irradiation. In rats that were pretreated with EGCG at a dose of 2.5 and 5 mg/kg, plasma homocysteine was significantly decreased when compared to the first group. The homocysteine levels were significantly decreased in the second and third groups as compared to the first group [101].

#### 2.1.3. Cinnamon

**Figure 3:**
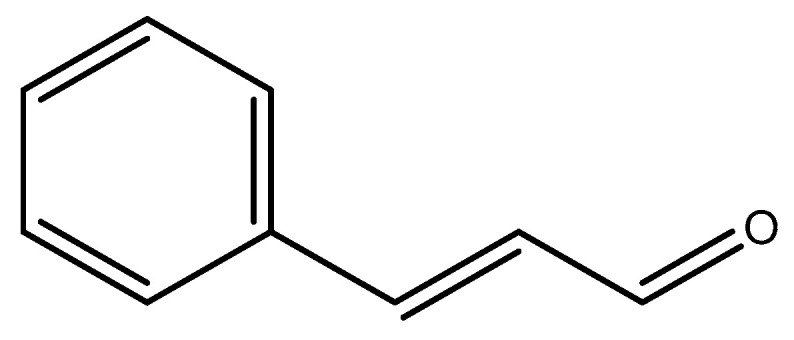
Chemical structure of cinnamaldehyde.

Cinnamon (*Cinnamomum verum* J.Presl), a plant from the Lauraceae family, is mostly used as a spice [102]. It is a herbal medicine used for conditions, such as flatulence, amenorrhea, diarrhea, toothache, fever, leukorrhea, common cold, and headache [103,104]. Cinnamon and its main component, Cinnamaldehyde (Figure 3), have insulin sensitizer, antioxidant, and anti-inflammatory properties [105,106]. This herbal medicine was also traditionally recommended for the treatment of impotency, frigidity, dyspnea, eye inflammation, vaginitis, cough, rheumatism, neuralgia, and CVDs [107]. A two-phase clinical trial conducted by Amin et al. was conducted on 48 male albino rats for 5–8 weeks. Initially, hypercholesterolemia (the addition of 1% cholesterol powder, 0.25% bile salts, and beef tallow in a percentage of 4% to basal normal diet for 15 days) was induced. Subsequently, during the treatment period (starting from the third week and continued for six weeks), the hypercholesterolemic rats were divided into three subgroups (12 rats per group) according to the type of treatment. These regimens included HCD, HCD + Atorvastatin (0.2 mg/kg body weight), and HCD + cinnamon (*C. zeylanicum* Blume) (20 mg/day/rat). It was showed that cinnamon extract could reduce hypercholesterolemia and modulate oxidative stress and homocysteine [108].

#### 2.1.4. Anthocyanin

The word anthocyanin is derived from two Greek words, plant (*Anthos*) and blue (*kianos*), which are the most important pigments in vascular plants [109]. Like chlorophyll, they are natural pigments that are non-toxic, water-soluble, and widely present in plant cells [110]. Anthocyanins are the most colorful compounds among the flavonoids that are responsible for different colors found in many fruits, vegetables, and flowers [111,112]. They play essential roles in the management of CADs due to their high antioxidant effect [113,114]. In one clinical trial, 20 healthy female volunteers were randomly assigned into two groups to receive a placebo beverage or cranberry juice (750 mL/day (3 × 250 mL), which contained 2.80 mg/L anthocyanins) for two weeks. In the end, the plasma total homocysteine remained unchanged [115].

#### 2.1.5. Garlic Extract

**Figure 4:**
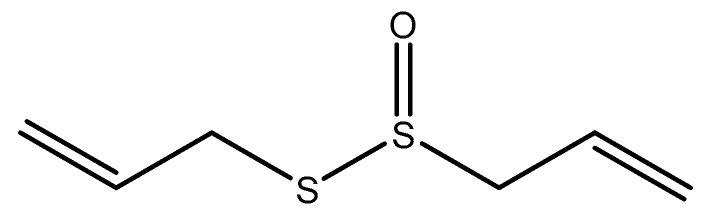
Chemical structure of allicin.

Garlic (*Allium sativum* L. Liliaceae) is a well-recognized medicinal plant. Several pharmacological implications of A. sativum and its organosulfur compounds, especially Allicin (Figure 4), include antibacterial, antiviral, antifungal, antiparasitic, anticancer, anti-inflammatory, and cardiovascular protective properties [116,117,118,119,120,121,122,123]. It has beneficial roles on dyslipidemia, which lowers the total cholesterol concentrations by approximately 10% and favorably alters HDL/LDL ratios. Additionally, it acts as a mild anti-hypertensive that reduces blood pressure by 5–7% [124]. A group of 60 subjects were randomly assigned to two groups: (I) intervention (received a daily capsule of aged garlic-extract (AGE) (250 mg) plus vitamin-B12 (100 μg), folic-acid (300 μg), vitamin-B6 (12.5 mg), and L-arginine (100 mg) for 12 months) and (II) placebo. Finally, a reduced level of homocysteine was observed in the intervention group [125]. In a study conducted by Budoff et al., 65 patients with an intermediate risk for CVDs were randomly allocated to two groups: (I) the daily administration of a capsule containing AGE (250 mg) plus Vitamin B12 (100 μg), folic acid (300 μg), Vitamin B6 (12.5 mg), and L-arginine (100 mg) or (II) placebo. After one year of treatment, the homocysteine level was decreased in the AGE group [126]. In another experiment, 40 rats were employed to receive one of the following diets for six weeks (four groups of 10 each): (I) AIN-93G folic-acid sufficient (2 mg/kg of diet); (II) AIN-93G folic-acid deficient; (III) AIN-93G folic-acid sufficient that was supplemented with AGE (4% of diet, wt:wt); and, (IV) AIN-93G folic-acid deficient supplemented with AGE. The results showed that the addition of AGE to the severely folate-deficient diet decreased the total plasma homocysteine concentration by 30% [127]. Based on a similar study, 23 patients with known CAD were randomly assigned to two groups: (I) received AGE (4 mL) or (II) received placebo for one year. Following intervention, the homocysteine levels showed no significant improvement [128]. In another randomized controlled trial (RCT), 30 postmenopausal women were randomly assigned to four groups: (1) placebo, (2) consumption of AGE (5 × 65 mg per week), (3) exercise (60% of maximum heart rate) and placebo, and (4) exercise and aged garlic extract. After 12 weeks, homocysteine was significantly decreased with intervention in the second and fourth groups [129]. Ried et al., conducted an RCT on 30 pediatric (aged 8 to 18 years old) patients who were administered 300 mg AGE (three capsules/ daily) or placebo. After eight weeks of treatment, no significant difference was observed in homocysteine levels between groups [130]. In a similar RCT, 88 uncontrolled hypertensive patients were given 1.2 gr of AGE powder+ 1.2 mg *S*-allylcysteine each day or placebo for 12 weeks. Following intervention, no significant change was detected in the homocysteine level in both groups [131].

#### 2.1.6. Ginseng

**Figure 5:**
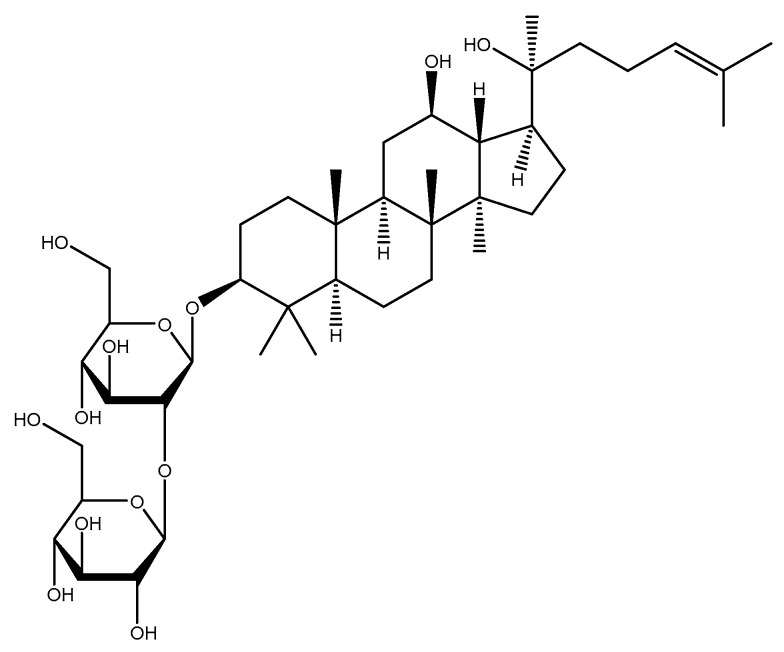
Chemical structure of ginsenoside Rg3.

Ginseng (*Panax ginseng* C.A.Mey., Araliaceae) is a medicinal plant with favorable pharmacological effects in cancer, diabetes, and CVDs, which also improves the immune system and central nervous system (CNS) function, relieves stress, and possesses antioxidant properties, and these benefits are mainly attributed to the presence of ginsenosides (Figure 5) [132,133,134,135]. A group of 40 wistar male rats were randomly assigned to one of the following four groups: (1st) control group (tap water), (2nd) Methionine (Met) (1 g/kg per day) and succinyl sulfathiazole (SSL) (0.5 g/kg per day), (3rd) ginsenosides total saponins (GTS) (Korean ginseng) (50 mg/kg every 12 h), and (4th) Met + GTS + SSL. The homocysteine levels were measured within 30 and 60 days of the intervention, and they were found to be reduced in the fourth group as compared to the second group, whereas the third group had no significant change when compared to the first group [136].

#### 2.1.7. Chlorella

Chlorella is a genus of approximately thirteen species of single-celled green algae belonging to the division Chlorophyta [137]. *Chlorella pyrenoidosa*, a single cell alga that is found in freshwater, has a rich content of chlorophyll and high concentration of nucleic acids, minerals, amino acids, dietary fiber, and vitamins. It has a strong cell wall that can only be digested by humans during a Dyno-Mill process. Studies showed that injection or oral intake of Chlorella following its cell wall destruction improves the immune function against infection and anti-cancer activity [138,139,140,141,142,143]. In study conducted by Merchant et al., 17 vegetarian/vegan (aged 26–57 years old) with a vitamin B12 deficiency were asked to add 9 gr/day of *C. pyrenoidosa* to their routine diet for 60 days. The findings reveled that the homocysteine level was reduced by an average of 10% [144].

#### 2.1.8. Ginger

**Figure 6:**
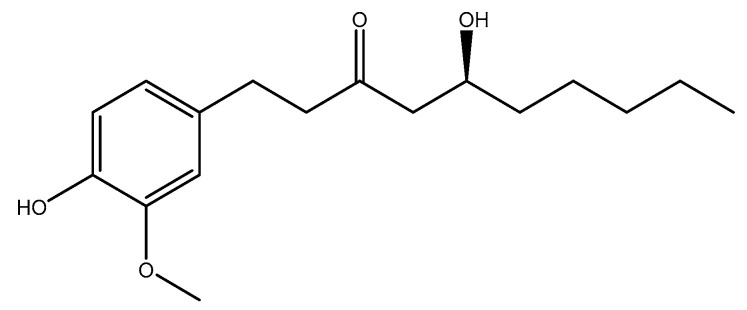
Chemical structure of [6]-gingerol.

Ginger (*Zingiber Officinal*e Roscoe, Zingiberaceae) is a part of a family of plants, including cardamom and turmeric. It has a strong aroma due to the presence of gingerols (pungent ketones) (Figure 6). The rhizome part of the plant is traditionally used in Asia and tropical areas for fever, common cold, digestive problems, among others. Ginger is an appetite stimulant, and it is categorized by the U.S. FDA as a food additive [145]. Its beneficial effects on nausea/vomiting and arthritis have been previously reported [146,147,148,149,150,151,152,153]. In one experiment, 24 male Wistar rats were randomized into three groups: (1) non-diabetic control (tap water), (2) non-treated diabetic (tap water), and (3) ginger (*Z*. *Officinale*) extract treated diabetic (50 mg/kg of hydroalcoholic ginger + tap water). After six weeks, the homocysteine level was notably enhanced in the second group when compared to the first group, whereas it was significantly declined in the third group as compared to the second group [154]. In another experiment by Akbari et al., 28 adult male Sprague Dawley rats were randomized into four groups: (1) control (2 mL/day corn oil), (2) ginger (*Z*. *Officinale*) (1 g/kg body weight daily), (3) ethanol (4 g/kg body weight daily), and (4) ethanol-ginger (*Z*. *Officinale*). After 28 days, the homocysteine level was significantly increased in the third group when compared to the first group, whereas it was significantly decreased in the fourth group as compared to the third group [155].

#### 2.1.9. Soy

**Figure 7:**
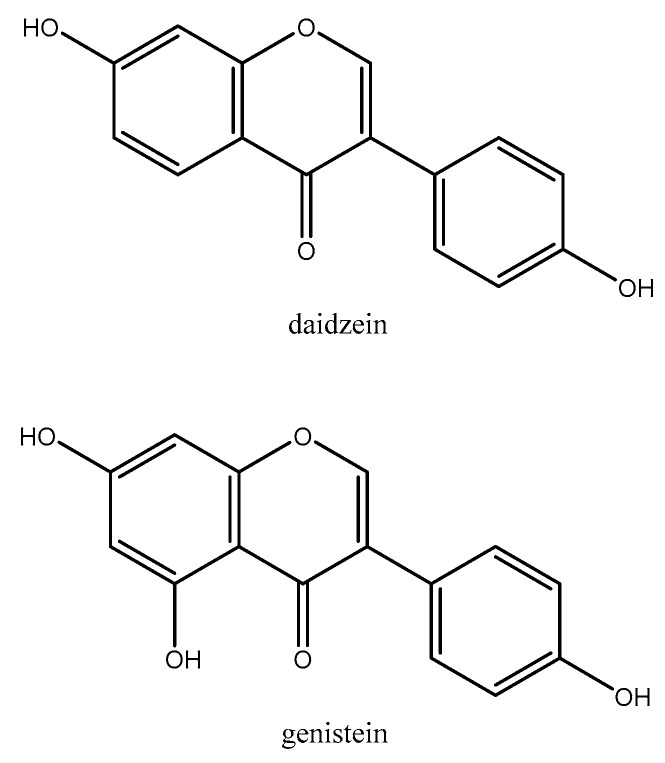
Chemical structures of daidzein and genistein.

Soybean (*Glycine max* (L.) Merr., Fabaceae) is a traditional plant that is native to East Asia, which is a good source of phytochemicals (e.g., isoflavones: daidzein, and genistein, Figure 7), fiber, and plant sterols [156,157,158]. Various studies have revealed its beneficial effects on blood lipids, CVDs, fertility, and menopause [159,160,161]. The result of a systematic review and meta-analysis (2016), which reviewed 19 randomized controlled studies, showed that Soy or isoflavones had no effect on homocysteine levels [162]. In a randomized cohort study, 87 healthy postmenopausal women were assigned to one of these groups: (1) 1200 Kcal diet + exercise or (2) 1200 Kcal diet + exercise + 200 mg of Glycine max (80 mg of soy isoflavone: 60.8 mg of genistein, 16 mg of daidzein, and 3.2 mg of glicitein) for six months. Following intervention, the homocysteine level remained unchanged in both of the groups [163]. A group of 117 patients with hypercholesterolemia were also divided into three groups to receive: 15 gr/day soy protein (containing SuproSoy from Solae, Saint Louis, MO, USA) and 25 gr/day soy protein or placebo. No change in homocysteine level as compared to the baseline was found in all groups [164]. Similarly, in a cross over study on 34 postmenopausal women, the participants received 26 ± 5 g/day of isolated soy protein (containing 44 ± 8 mg isoflavones per day) or 26 ± 5 g/day of milk protein isolate for six weeks each and two weeks washout. The homocysteine level was not different between the two groups [165]. Forty patients on peritoneal dialysis were studied in a clinical trial; two groups were, as follows: received 28 gr/day of textured soy flour (contained 14 g of soy protein) or followed their regular diet for eight weeks. The concentration of homocysteine had no significant difference between the two groups [166]. In another randomized crossover study on forty-one hyperlipidemic men and postmenopausal women, three groups were assigned to receive one of these regimens: (1) a low-fat dairy control diet, (2) low-isoflavone soy food diet (10 mg isoflavones/day), and (3) high- isoflavone soy food diet (73 mg isoflavones/day). Intervention was conducted for 3* one months, allowing a two-week washout period between interventions. It was showed that the homocysteine level was lower in both isoflavones groups when compared to the control group [167]. A total of 55 postmenopausal women (aged 42–72 years) in a double blind clinical trial were randomly recruited to receive one of the four soy protein isolate treatments (40 g/d): (1st) normal phytate and isoflavone (Phytate: 0.78 g and Aglycone isoflavones: 84.6 mg), (2nd) normal phytate and low isoflavone (Phytate: 0.64 g and Aglycone isoflavones: 1.2 mg), (3rd) low phytate and normal isoflavone (Phytate: 0.22 g and Aglycone isoflavones: 85.5 mg), and (4th) low phytate and isoflavone (Phytate: 0.22 g and Aglycone isoflavones: 1.2 mg). After six weeks, the homocysteine levels were significantly reduced in soy protein normal phytate groups, whilst no significant change was detected in the soy protein normal isoflavone groups [168]. In another recent randomized crossover study, 89 postmenopausal women were randomly assigned to consume (1) two fruit cereal bar,s each including 25 mg soy isoflavons (genistein:daidzein ratio of 2:1) or (2) only two fruit cereal bars with no isoflavones. The intervention was conducted for eight weeks with an eight-week washout. The results showed that homocysteine did not change in both groups [169]. In a recent double-blind RCT, 38 postmenopausal women complaining of insomnia were asked to either consume 80 mg/day of isoflavones (60.8 mg of genistein, 16 mg of daidzein, and 3.2 mg of glicitein) daily or placebo for four months. Following intervention, the homocysteine showed no significant change in both groups [170]. Similarly, 30 female wild-type mice were randomly allocated to three groups: (I) the control group (modified standard maintenance chow), (II) soy group (55% total energy), or (III) casein group (55% total energy). After 12 weeks, the homocysteine level was higher in casein group when compared to the control group, while the homocysteine level remained the same in the soy and control groups [171]. A total of 24 adult female Sprague Dawley rats were also randomized into four groups: (1) 2% cholesterol diet (2) 2% cholesterol diet + fresh soy oil, (3) 2% cholesterol diet + one-heated soy oil, and (4) 2% cholesterol diet + five-time-heated soy oil. After four months, 2% cholesterol diet + fresh soy oil significantly reduced the homocysteine level when compared to the others [172].

#### 2.1.10. Emblica Officinals (Amla)

**Figure 8:**
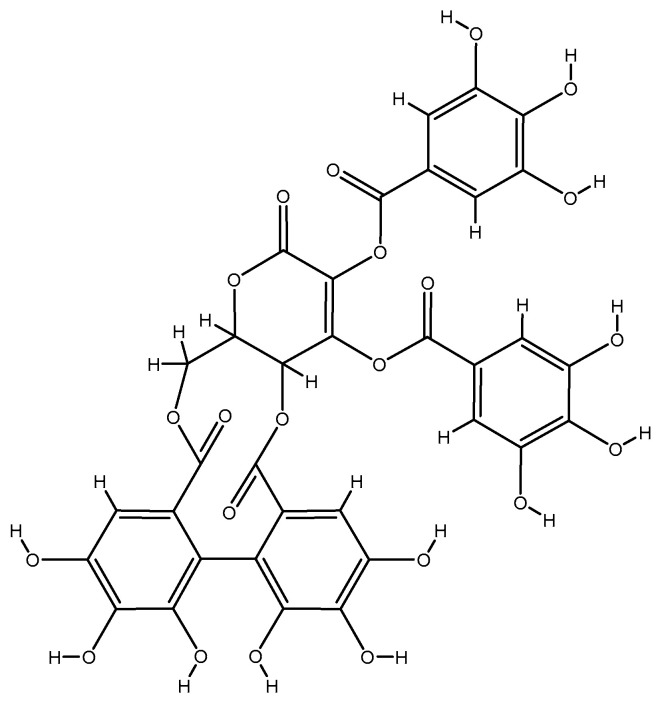
Chemical structure of emblicanin A.

*Emblica officinalis* L. (Phyllanthaceae), which is known as Indian Gooseberry or Amla, is a fruit that has high levels of ascorbic acid (from 1100 to 1700 mg/100 g of fruit extract) and a high density of ellagitannins including emblicanin A (37%) (Figure 8), emblicanin B (33%), punigluconin (12%), and pedunculagin. Amla is often consumed as a functional food due to its physiological features, such as radioprotection [173,174,175], antioxidant activity [176,177,178,179,180], hepatoprotection [181,182,183,184], cytoprotection [185,186], and hypolipidemic effects [176,187,188,189]. In a recent double-blind RCT, 98 patients affected with dyslipidemia were asked to either consume 500 mg capsule of Amla extract (from Indian gooseberry) each day or placebo for 12 weeks. Following intervention, the homocysteine level did not change significantly in both groups [190]. Similarly, 17 uremic patients were selected to receive Amla extract tablets (300 mg, 50% dextrin + 50% amla extract) four times a day. Based on the findings, the homocysteine level did not change after four months of intervention [191].

#### 2.1.11. Nuts

Nuts have scant amounts of bioactive compounds (e.g., phytosterols, unsaturated FAs, fiber, protein, vitamins, calcium, magnesium, sodium, and potassium) and they are a good source of antioxidants [192,193,194]. In a study on 15 hypercholesterolemic (serum cholesterol level > 200 mg/dl) adult males (aged 33–59 years), the patients received hazelnut (*Corylus avellana* L., Betulaceae) enriched diet (control diet (low-fat, low-cholesterol, and high-carbohydrate) + 40 g/d hazelnut) for four weeks directly after four weeks of a control diet. Following intervention, no change in homocysteine level was detected between the groups [195]. In a two-phase single blind crossover study on 67 patients (serum total cholesterol > 5.2 mmol/L), the participants first consumed low-fat, low-cholesterol diet for six weeks. On the second phase, two groups were randomly selected to either consume 64 g/d walnut (*Juglans regia* L., Juglandaceae) with their diet or continue the same diet for six weeks. Finally, all of the patients were crossed over into the opposite treatment arm for another six weeks. The findings reveled no statistically significant change on the homocysteine levels [196].

#### 2.1.12. Olive Oil

Olive oil, which is known as the elixir of health and youth, consists almost one-third to two-thirds of the fat used in the Mediterranean diet [197,198,199,200,201]. Many studies have shown its beneficial effects in improving heart diseases or cancer [202,203,204,205]. In one clinical trial, 121 obese (BMI ≥ 35 kg/m^2^) adults (aged 18–65 years) randomly received one of these three diets: (I) 52 mL/d extra virgin olive oil (EVOO), (II) traditional Brazilian diet (DieTBra), or (III) 52 mL/d EVOO + (DieTBra). After 12 weeks, the homocysteine levels reduced in the EVOO group by nearly 10% (mean), whilst the homocysteine levels did not change significantly between groups two-by-two [206].

### 2.2. Phytochemicals

#### 2.2.1. Berberine

**Figure 9:**
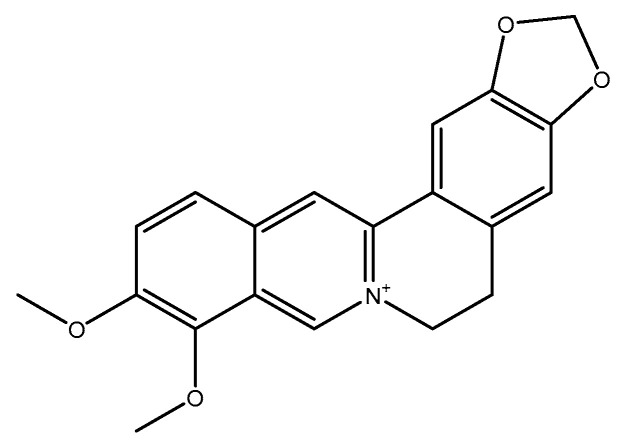
Chemical structure of berberine.

Berberine, a benzylisoquinoline alkaloid (Figure 9), is an active constituent in numerous medicinal plants with many pharmacological properties. It has been largely used in Ayurvedic and Chinese medicine for its antimicrobial, antiprotozoal, antidiarrheal, and antitrachoma activities. Several clinical and preclinical studies have indicated the promising effects of berberine on metabolic, neurological, and cardiological disorders [207,208,209,210,211,212]. In a study, healthy male rats (weighing 190–210 g) randomly received a standard diet or a high-fat diet (HFD) for 24 weeks. After eight weeks of feeding, rats that were fed with HFD were randomly assigned into two groups: (I) berberine (extracted from *Coptis chinensis* Franch.) (200 mg/kg/day) or (II) vehicle by gavage for 16 weeks (*n* = eight per each group). The results showed that the berberine consumption led to a significant reduction in serum homocysteine by about 60% when compared to the vehicle in rats fed with a HFD [213]. In a double-blind RCT, 31 diabetic patients were randomly assigned into two groups to receive *Berberis vulgaris* L. fruit extract (3 g/d) (which contains the berberine alkaloid) or placebo for three months. No significant change was observed in the serum homocysteine level between the two groups [214].

#### 2.2.2. Curcumin

**Figure 10:**
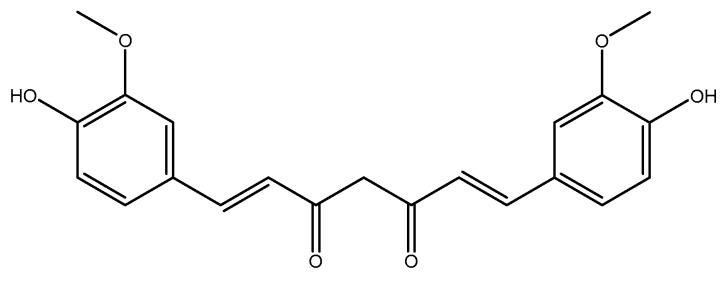
Chemical structure of curcumin.

**Figure 11:**
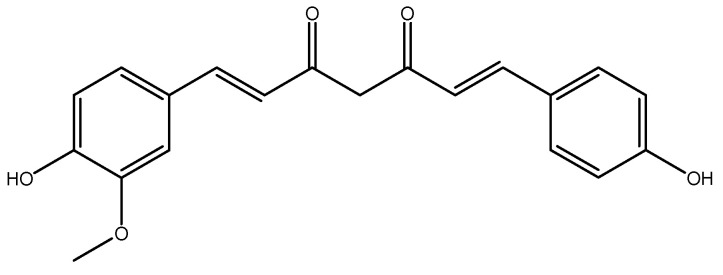
Chemical structure of Demethoxycurcumin.

**Figure 12:**
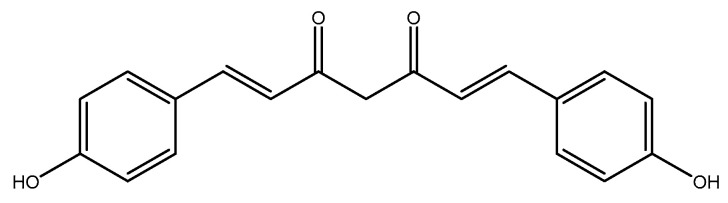
Chemical structure of Bisdemethoxycurcumin.

Curcumin, a turmeric-derived polyphenol (*Curcuma longa* L., Zingiberaceae) (Figure 10), is known for its safety and medicinal properties against a variety of diseases [215,216,217,218,219,220,221,222,223,224]. It also has beneficial effects in metabolic syndrome and obesity [225,226]. In a recent double-blind RCT, 22 obese men were administered a 500 mg curcumin supplement (193 mg of curcuminoids in the form of 81.8% curcumin, 15.3% demethoxycurcumin (C20H18O5) (Figure 11), and 2.8% bisdemethoxycurcumin(C19H16O4) (Figure 12) were infused into 60% soluble fiber from fenugreek to improve bioavailability) or placebo supplement (comprised of equal parts of soluble fiber from fenugreek) every day for 12 weeks. After intervention, the plasma homocysteine concentration was significantly reduced in the intervention group when compared to the placebo group [227]. In a clinical trial, female Wistar–Furth rats were randomly divided into low *ω*-3 PUFA (LFO, *n* = 12) and high *ω*-3 PUFA (HFO, *n* = 12) groups and they were further divided into three subgroups: LFO or HFO (controls), LFOE or HFOE (LFO or HFO plus ethanol, 35% of dietary calories derived from ethanol), and LFOEC or HFOEC (LFOE or HFOE supplemented with curcumin 150 mg/kg body weight/day). All of these groups were pair-fed for eight weeks. Curcumin caused a significant increase in homocysteine thiolactonase activities as compared to the high *ω*-3 PUFA and ethanol groups [228]. In a group of fifty healthy men who were randomly selected, a two month consumption of biscuits with a bioactive complex, such as organic selenium (115 mg), quercetin (6 g dried selenized onion), curcumin (1.3 g curcuma), and catechins (2 g green tea), was associated with decreased homocysteine levels [229]. In a clinical trial on 32 adult male Wister rats, four groups were selected: (1) control group (no injection), (2) vehicle of homocysteine (2 μmol/μL), (3) vehicle of curcumin (50 mg/kg), and (4) homocysteine-curcumin group. Curcumin was injected intraperitoneally once daily for 10 days, beginning five days prior to homocysteine intracerebroventricular injection. In the end, curcumin significantly reversed the behavioral and biochemical changes caused by exposure of homocysteine in the control mice. Similar to homocysteine, curcumin could be considered to be a therapeutic agent in preventing the progression of neurotoxicity [230]. A total of 50 female Sprague–Dawley rats (weighing 220–250 gr) were randomly divided to the following groups: (1) control (0.3 mL of the vehicle), (2) sham (0.3 mL of the vehicle + surgery stress), (3) danazol treatment (7.2 mg/kg BW), (4) curcumin treatment (48 mg/kg BW), and (5) test (0.3 mL of the vehicle). After four weeks of treatment, no significant difference was observed between all groups [231].

#### 2.2.3. Resveratrol

**Figure 13:**
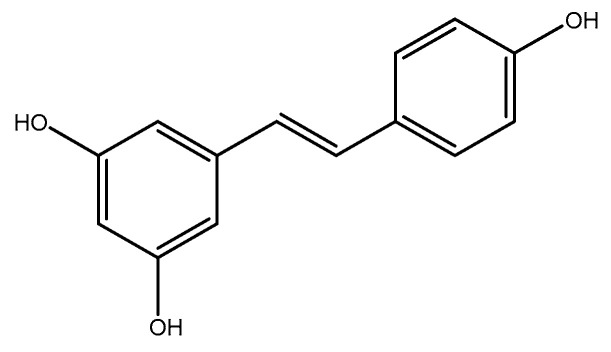
Chemical structure of resveratrol.

Resveratrol (3,5,4′-trihydroxy-*trans*-stilbene) (Figure 13), a natural polyphenol that is found in numerous fruits and vegetables, has several properties, including anti-aging, anticancer, anti-inflammatory, and antidiabetic. Although the positive effects of resveratrol against selected cardiovascular risk factors have been a subject of debate over recent years [232,233], there are numerous effects against oxidative stress, apoptosis, mitochondrial dysfunction, endothelial dysfunction, and angiogenesis that might still support the potential role of this phytochemical in the prevention of CVDs [234,235,236]. A total of 24 rats were randomly assigned to three groups: (I) control group (received standard rat food), (II) homocysteine group (received 1 g/kg bodyweight/day methionine in drinking water), and (III) homocysteine + resveratrol group (received same amount of methionine + 20 mg/kg/day resveratrol intraperitoneally). After 30 days, the results indicated that the plasma homocysteine level was significantly reduced in a group that was treated with resveratrol [237]. In a study conducted by Noll et al., the mice were randomly divided into four groups and maintained on the following diets for three months: (I) control group (received standard rodent diet), (II) high methionine (received standard diet plus 0.5% L-methionine (36 mg/day) in drinking water), (III) methionine/resveratrol (received high-methionine diet with 0.001% trans-resveratrol (50 µg/day) in the last month, and (IV) resveratrol (received standard diet with 0.001% trans-resveratrol in the last month). The findings showed that plasma total homocysteine concentration in high methionine group was four times higher than the control group. In the methionine/resveratrol group, plasma total homocysteine levels were non-significantly increased when compared to the methionine group. However, in methionine/resveratrol group plasma homocysteine levels were 1.3 times higher as compared to the methionine group. Additionally, in the resveratrol group, the homocysteine levels were significantly increased by 1.7 (uM) when compared to the control group. Based on these results, resveratrol had detrimental effects on homocysteine levels [238]. Similarly, an experiment was performed on 30 female rats that were randomly allocated to three groups (I) control group, (II) potassium bromate group (KBrO_3_ 80 mg/kg), and (III) resveratrol (33 mg/kg four times a week) + KBrO_3_ (80 mg/kg). These treatments were continued for five weeks. The findings demonstrated that the serum homocysteine levels in the resveratrol+ KBrO3 group were significantly lower than the control group [239]. According to a study conducted by Schroecksnadel et al., peripheral blood mononuclear cells (PBMC) were isolated from healthy volunteer blood donors by density centrifugation. In order to examine the effects of resveratrol on PBMCs, the cells were either pre-incubated with 10–100 mM resveratrol or stimulated with mitogens after 30 min., or resveratrol was added 2 h after stimulation. The cells were incubated at 37°C in 5% CO_2_ for 72 h and supernatants were harvested by centrifugation and then frozen at –208 °C until measurement. The results showed that unstimulated PBMCs produced small amounts of homocysteine and pretreatment of unstimulated cells with 10–100 mM resveratrol only slightly decreased the homocysteine production of resting cells [240].

### 2.3. Other

#### Soluble Fiber

Water-insoluble fibers enhance the stool bulk and improve bowel movements. The viscous or gel-forming fibers (e.g., gums, mucilages, pectins, algal polysaccharides, some hemicelluloses, and some storage polysaccharides) are water soluble. Good sources of water-soluble fiber include oats, dried beans, barley, some vegetables, and fruits [241,242]. The positive effects of soluble fibers on cardiac diseases, hyperlipidemia, diabetes mellitus, insulin resistance, and obesity are well known [243]. In a double-blind randomized parallel controlled study, 29 overweight men (aged 20–69 years, BMI: 25–35 kg/m^2^) were asked to consume 3 g soluble fiber every day or placebo for 12 weeks along with a carbohydrate restricted diet and standard daily multivitamins. In the end, the homocysteine level did not significantly increase in the fiber group as compared to the placebo group [244]. A total of 119 subjects were randomly recruited to two groups: (I) multivitamin and 4g soluble fiber or (II) placebo. After eight weeks of intervention, the homocysteine levels were significantly reduced in the fiber blend group when compared to the placebo group [245].

## 3. Conclusions and Future Perspective

This review demonstrated that medicinal plants and herbal bioactive compounds have promising effects on reducing the homocysteine levels. According to the clinical trials, resveratrol and curcumin had favorable effects on serum homocysteine levels, whilst the results regarding other compounds were inconclusive (Table 1). In some of the previous studies, homocysteine was evaluated as a secondary outcome, and its effects on treatment or prevention of diseases were not elucidated. Moreover, factors, which included small sample size, diverse population in terms of age, being healthy or having underlying chronic diseases, different herbs with dissimilar dosages, and different duration of interventions, made it difficult to draw a strong evidence-based conclusion. Based on almost all animal studies, medicinal plants showed promising effects on homocysteine. Black and green tea, cinnamon, resveratrol, curcumin, garlic extract, ginger, and soy significantly reduced the homocysteine concentrations (Table 2). Altogether, this review highlighted the beneficial effects of medicinal plants as natural, inexpensive, and accessible agents without any considerable adverse effects on homocysteine levels. Nevertheless, the results of the clinical trials were not uniform, which suggested that more well-designed comprehensive clinical trials are warranted.

## Figures and Tables

**Table 1:** The effects of herbal bioactive compounds on homocysteine levels according to clinical trials.

Number	Author, Year	Agent	Dose per Day	Treatment Duration	Subjects	Main Outcome(s)	Final Effects of Nutraceuticals on Homocysteine Level
1	Hodgson et al., 2007 [85]	Black Tea	2.2 g tea leaves	3.5 h	Adults with CAD	Black tea significantly increased plasma homocysteine levels	↑
2	Hodgson JM et al., 2003 [86]	Black Tea	2 g tea leave/250 mL boiled water	12 weeks	Healthy subjects	Black tea did not significantly alter mean homocysteine concentrations	No effect
3	Olthof MR et al., 2001 [87]	Black tea	4 g black tea	4 weeks	Healthy subjects	Black tea raised total plasma homocysteine concentrations	↑
4	Duthie et al., 2006 [115]	Anthocyanins	750 mL/day cranberry juice (2.80 mg/L anthocyanins)	2 weeks	Healthy volunteer females	Cranberry juice had no effect on plasma homocysteine levels	No effect
5	Ahmadi N et al., 2013 [125]	Garlic extract	Aged garlic extract (AGE) 250 mg + B12 100 μg + B9 300 μg + B6 12.5 mg + L-arginine 100 mg	12 months	Healthy subjects	Garlic extract plus other supplements reduced homocysteine level	↓
6	Budoff MJ et al., 2009 [126]	Garlic extract	AGE 250 mg + B12 100 μg + B9 300 μg + B6 12.5 mg + L-arginine 100 mg	1 year	Healthy subjects	Garlic extract plus other supplements reduced homocysteine level	↓
7	Budoff MJ et al., 2004 [128]	Garlic extract	4 mL	1 year	Patients with CAD	Garlic extract did not significantly improve homocysteine level	No effect
8	Seo DY et al., 2012 [129]	Garlic extract	5 × 65 mg per week	12 weeks	30 postmenopausal women	Homocysteine was significantly decreased	↓
9	McCrindle BW et al., 1998 [130]	Garlic extract	3 × 300 mg per day	8weeks	30 pediatric (8 to 18 years old)	No significant difference between the groups	No effect
10	Ried K et al., 2016 [131]	Garlic extract	1.2 g powder per day + 1.2 mg *S*-allylcysteine	12 weeks	88 uncontrolled hypertensive patients	No significant differences between the groups	No effect
11	Merchant RE et al., 2015 [144]	Chorella	9 g per day	60 days	17 vegetarian or vegan	Homocysteine level decreased by an average of 10%	↑
12	Llaneza P et al., 2011 [163]	Soy	80 mg of soy isoflavone	6 months	87 healthy postmenopausal women	No change in both groups	No effect
13	Høie LH et al., 2005 [164]	Soy	15 or 25 g of soy protein	8 weeks	117 Hypercholesterolemic patients	No change in all groups	No effect
14	Greany K et al., 2008 [165]	Soy	26 ± 5 g/day of soy isolated protein	2 × 4 weeks + 2 weeks washout	34 postmenopausal women	No change between both groups	No effect
15	Imani H et al., 2009 [166]	Soy	28 g/day of soy textured soy flour	8 weeks	40 peritoneal dialysis patients	No change between groups	No effect
16	Jenkins DJ et al., 2002 [167]	Soy	Low fat dairy food control diet, with low or high isoflavone soy food diets	3 × 1 months + 2 weeks washout	41 hyperlipidemic men and postmenopausal women	Homocysteine was lower in both isoflavone groups	↓
17	Hanson LN et al., 2006 [168]	Soy	40 g/day soy protein isolate	6 weeks	55 postmenopausal women	homocysteine was significantly reduced in soy protein normal phytate group, while no significant change was detected in soy protein normal isoflavone group	soy protein normal phytat: ↓ soy protein normal isoflavone: no effect
18	Reimann M et al., 2006 [169]	Soy	50 mg soy isoflavone	8 weeks	89 postmenopausal women	Homocysteine level did not change in both groups	No effect
19	Brandao LC et al., 2009 [170]	Soy	80 mg soy isoflavone	4 months	38 postmenopausal women	Homocysteine level did not change in both groups	No effect
20	Upadya H et al., 2019 [190]	Amla	500 mg capsule of Amla extract	12 weeks	98 patients with dyslipidemia	Homocysteine level did not change significantly between the groups	No effect
21	Chen T-S et al., 2009 [191]	Amla	amla extract tablets (300 mg, 50% dextrin + 50% amla extract) four times a day	4 months	17 uremic patients	Homocysteine level did not change in subjects	No effect
22	Mercanlıgil S et al., 2007 [195]	Nut	40 g/day hazeinut	8 weeks	15 hypercholesterolemic patients	Homocysteine level did not change between the groups	No effect
23	Morgan J et al., 2002 [196]	Nut	64 g/day walnut	18 weeks	67 patients (serum total cholesterol > 5.2 mmol/L)	No statically significant effects were observed	No effect
24	Rodrigues APdS et al., 2020 [206]	Olive oil	52 mL/d EVOO	12 weeks	121 obese adult subjects	Homocysteine levels did not change significantly between the groups	No effect
25	Shidfar F et al., 2012 [214]	Berberis vulgaris Fruit Extract	3 g/d	3 months	Diabetic patients	Berberine did not significantly alter mean serum homocysteine concentration	No effect
26	Campbell MS et al., 2019 [227]	Curcumin	500 mg	12 weeks	Obese men	Homocysteine was significantly reduced in the curcumin group	↓
27	Madaric A et al., 2013 [229]	Curcumin	100 g of biscuits per day with 1.3 g curcuma	2 months	Healthy men	Curcumin significantly decreased homocysteine level	↓
28	Schroecksnadel K et al., 2005 [240]	Resveratrol	1–100 µg	72 h	Healthy voluntary blood donors	Pretreatment of unstimulated cells with 10–100 mM resveratrol only slightly decreased homocysteine production in the resting cells	↓
29	Wood RJ et al., 2006 [244]	soluble fiber	3 g soluble fiber	12 weeks	29 overweight men	Homocysteine level did not significantly increase in fiber group compared to the placebo group	No effect
30	Sprecher DL et al., 2002 [245]	soluble fiber	4 g soluble fiber	8 weeks	119 subjects	Homocysteine levels significantly reduced in fiber blend group compared to the placebo group	↓

↑: Increasing effect; ↓: decreasing effect.

**Table 2:** The effect of herbal bioactive compounds on homocysteine levels based on animal studies.

Number	Author, Year	Agent	Dose per Day	Treatment Duration	Animals	Main Outcome(s)	Final Effects of Nutraceuticals on Homocysteine Level
1	San Cheang et al., 2015 [84]	Black Tea extract	15 mg/kg/day	2 weeks	Rats	Black tea extract significantly reduced plasma homocysteine levels	↓
2	El-Missiry MA et al., 2018 [101]	Green tea	2.5 or 5 mg/kg body weight EGCG	3 days	Adult male Wister rats	EGCG at a dose of 2.5 and 5 mg/kg significantly decreased plasma homocysteine	↓
3	Amin KA et al., 2009 [108]	Cinnamon extract	20 mg/day/rat	5–8 weeks	Male rats	Cinnamon extract reduced homocysteine levels	↓
4	Yeh YY et al., 2006 [127]	Garlic extract	4% of diet	6 weeks	Rats	Garlic extract significantly reduced homocysteine level	↓
5	Kim JH, 2019 [136]	ginsenoside	50 mg/kg every 12 h	60 days	40 wistar male rats	Met reduced plasma Homocysteine level, whereas GTS did not affect basal plasma levels	GTS alone: No effect Met: ↓
6	Ilkhanizadeh B et al., 2016 [154]	ginger	50 mg/kg body weight daily	6 weeks	24 male Wistar rats	Significant decrease in homocystein level was found in the ginger extract-treated diabetic group	↓
7	Akbari A et al., 2017 [155]	ginger	1 g/kg body weight daily	28 days	28 adult male Sprague-Dawley	In ginger-ethanol group, ginger improved antioxidant enzymes’ activity and reduced tHcy and MDA compared to the ethanol group	↓
8	Snelson M et al., 2017 [171]	Soy	55% total energy	12 weeks	30 female wild-type mice	homocysteine level was the same in soy and control group	No effect
9	Adam SK et al., 2008 [172]	Soy	Fresh soy oil One-heated soy oil Five-time-heated soy oil	4 months	24 adult female Sprague Dawley rats	fresh soy oil significantly reduced homocysteine level compared to the other groups	↓
10	Chang X-x et al., 2012 [213]	Berberine	200 mg/kg/day	24 weeks	Healthy male rats	Serum homocysteine level was significantly decreased after berberine consumption in rats fed with a high-fat diet	↓
11	Varatharajalu R et al., 2016 [228]	Curcumin	150 mg/kg body weight/day	8 weeks	Female Wistar-Furth rats	Curcumin significantly increased homocysteine thiolactonase activity	↓
12	Mansouri Z et al., 2012 [230]	Curcumin	50 mg/kg	10 days	Adult male Wister rats	Investigated the neuroprotective effects of curcumin against homocysteine neurotoxicity	↓
13	Jelodar G et al., 2019 [231]	Curcumin	48 mg/kg	4 weeks	female Sprague-Dawley rats	no significant difference was observed between all groups	No effect
14	Koz ST et al.2012 [237]	Resveratrol	20 mg/kg/day	30 days	Rats	Resveratrol significantly reduced plasma Homocysteine levels	↓
15	Noll C et al., 2009 [238]	Resveratrol	50 µg/day	3 months	Mice	Resveratrol significantly increased homocysteine levels compared to the control group	↑
16	Yilmaz Ö et al., 2007 [239]	Resveratrol	33mg/kg four times per week	5 weeks	Old female rats	Resveratrol significantly decreased homocysteine levels	↓

↑: Increasing effect; ↓: decreasing effect.

## Data Availability

There is no raw data associated with this review article.
